# New York Letter

**Published:** 1879-03

**Authors:** 


### Domestic Correspondence.

---

### ARTICLE XIV.

### NEW YORK LETTER.

NEW YORK CITY, Feb. 10, 1879.

GENTLEMEN: The over-crowded condition of New York has long caused it to have the highest death-rate of any American city, although the Board of Health is a very efficient one, and since its establishment has diminished the mortality considerably. This winter, however, it is likely to be larger than usual, owing to the great prevalence of scarlet fever. There have been and are now between two and three hundred cases every week, while the number last year was only about eighty. The cases have often been of a very malignant type, and in some weeks more than fifty per cent. have died. It is impossible to say what has brought on the epidemic. The public schools have disseminated it but slightly, although many of the buildings are ill-ventilated and unhealthy. A health officer visits each one every morning, reads off the localities where there are scarlet-fever cases, and if any of the children live in that locality they are excluded from school.

Diphtheria in this city, as in others, follows a regular line of increase or decrease. The number of cases here slowly increased until 1873, when it reached its maximum; since then it has been steadily diminishing. There are perhaps ten cases a week less this year than there were last.

The medical schools have been very flourishing. There are now probably over fifteen hundred medical students in the city—a number larger than in all England a year ago. Of the three large colleges, the University Medical College and the College of Physicians and Surgeons have had a considerable increase in students, while there has been a slight decrease at Bellevue Hospital Medical College. Each one of these institutions has its particular advantages, and it is not a matter of great importance which is chosen by the aspirant for a degree. The College of Physicians and Surgeons is the most "aristocratic" and has a better educated and better appearing class of students. Its strong point is its gynæcology, although in chemistry, surgery and pathology it also has perhaps the best men. The University gives the student more for his money, and boasts of its Loomis, who is the best teacher of medicine in the city. Bellevue makes much capital out of its name, and shrewdly words its announcements so that one might think it had a monopoly of Bellevue Hospital. It does get its students into that institution rather more than the other colleges, but it has no more claim upon it than they and but few more clinics there.

There are two female medical colleges in the city which have been vainly struggling for a respectable position. They continue to be feeble, partly because there are two and their forces are divided, partly because there isn't a supply of medical students. After examining a catalogue of one of these colleges, nothing can seem more ridiculous than an outcry against the intolerance and narrow-mindedness which excludes women from a medical education. There are in the catalogue the names of over thirty instructors, most of them well-known and eminent in their profession. And there are, on the other hand, some half-dozen grim girl graduates. Two professors to each pupil! Truly this is not oppression. It seems rather like meeting with magnificent liberality the Galenic aspirations of the female mind. The students are admitted to the hospital clinics, and eight or ten, it must be confessed rather scraggy-looking young women, may generally be seen upon the benches.

The hospitals and medical education here are almost entirely in the hands of the Regulars. There is one city hospital under homœopathic control, which treats chronic cases and the refuse of other hospitals. Amongst practitioners there are a number of wealthy and fashionable homœopaths—who have dropped most of their homœopathy—and who minister to the therapeutical idiosyncrasies of the ladies.

But New York's pet institution is its Woman's Hospital. Ten years ago the fashionable thing was obstetrics and Fordyce Barker; now it is gynæcology and T. Gaillard Thomas. The Woman's Hospital, as its annual report proudly asserts, is the only institution of its kind in the country, being devoted entirely to gynæcology. It is supported by endowments and subscriptions. The sum of $1,000 entitles one to have his or her name engraved upon a marble slab, which already proclaims the *aeternum decus* of dozens of philanthropists in the halls of the hospital building. The supporters and subscribers are amongst the *élite* of the city, and aristocracy turns its eleemosynary attention to leucorrhœal disturbances with a charming enthusiasm.

The hospital consists now of two separate four-story buildings, with a cottage for ovariotomies. Each building accommodates fifty or sixty patients. There are some free beds, but most of the patients pay from $4 to $15 per week. There are three wards in each building, one on each floor, besides private rooms. These wards are neat and comfortable, but without any elaborate decorations or appliances. Each bed is surrounded by curtains, a convenient arrangement on account of the necessity for frequent examinations. The hospital is a much-governed and much-visited institution. It has a board of governors, a board of lady supervisors, and a medical board and staff, the latter amounting in all to twenty-five for the hundred patients in the buildings. There is, however, an out-patient department which engages some of the medical officers. The character of the hospital is essentially surgical, and very fine results have been obtained in some directions. Operations upon the cervix and perineum are very popular, and almost uniformly successful. During the twenty-five years of the hospital's existence, ending a year ago, fifty-two ovariotomies were performed, of which one-half died. The per cent. of recoveries has lately been immensely increased. Since last October there have been a great many operations, some fifteen or twenty, without a death, although some of the cases were very bad ones. This record was interrupted only a short time ago. A woman entered the hospital in a very weak condition, with a large abdominal tumor. This tumor had a characteristic fluctuating feel, and seemed to be undoubtedly cystic. There was some doubt, however, as to whether it was ovarian or uterine. The woman failed to improve in her general condition, and operation was undertaken as a last resort. The usual short incision was made, and a tumor of immense size, but without adhesions discovered. A large trochar was pushed into it, but withdrew no fluid. The incision had therefore to be extended to the ensiform cartilage, in order to get out the tumor. The pedicle was short and thick, requiring the largest clamp to hold it. After clamping, the tumor was cut off, the stump ligatured and sewed into the abdominal wound, and the clamp removed. In the pedicle was an artery nearly as large as the femoral. The tumor itself proved to be a solid fibroid, probably uterine, and which had appeared at the very last moment, even to the experienced touch of Dr. Thomas, to be cystic. The woman failed to recover from the shock of the operation.

It is impossible not to admire the thoroughness with which every precaution is taken to secure the success of this climax of surgical art. There is a small, isolated wooden building erected especially for the operation and immediate after-treatment. At the time of operation the temperature is raised to that of the body. The antiseptic treatment was used, but has been generally abandoned as unnecessary or worse. There is a profusion of assistants, and of instruments for every possible emergency. With all these provisions, and with the high reputation which its recent great successes have gained, it is natural that ovarian tumors from the regions all around should gravitate toward the Woman's Hospital. The number of operations, as well as the per cent. of recoveries, is increasing every year, and at the present rate we can almost predict a time when the abdomen will be opened here with as little anxiety as an abscess.

